# Perceived difficulty of upwind shouting is a misconception explained by convective attenuation effect

**DOI:** 10.1038/s41598-023-32306-z

**Published:** 2023-03-31

**Authors:** Ville Pulkki, Rapolas Daugintis, Timo Lähivaara, Aleksi Öyry

**Affiliations:** 1 Acoustics Lab, Department of Information and Communications Engineering, Aalto University, 02150 Espoo, FI Finland; 2 Department of Technical Physics, University of Eastern Finland, 70211 Kuopio, FI Finland

**Keywords:** Acoustics, Computational science

## Abstract

It is a common thought that in windy conditions the voice of a shouter emanates towards the upwind with lower strength than towards the downwind. Contradicting with this, acoustics literature states that a source emanates with a higher amplitude against the upwind direction in comparison with the downwind direction, which is known as the convective amplification or attenuation effect. This article shows that the discrepancy arises because shouters receive their own voice at their ear canals worse when facing against the upwind direction than in the corresponding down-wind case. When shouting upwind, the ears are situated downwind from the mouth, and the strength of one’s own voice decreases in the ears due to the convective attenuation effect depending on frequency, making the shouter believe that it is more difficult to shout against the wind. This is shown by computational simulations and real measurements using models of a human shouter with simplified geometries.

## Introduction

In common language, the expression “shouting into the wind” refers to a fruitless attempt to communicate one’s thoughts when nobody can hear or understand them, which implies that, in general, people find shouting against wind somehow troublesome and ineffective. A common interpretation of the physical situation is that the “wind carries” the sound towards the downwind direction and prevents the emanation towards the upwind direction. However, the interpretation is wrong, since the direct effect of wind flow to emanating sound wave fronts is quite minor: the speed of sound is > 300 m/s, while the speed of wind is usually only < 20 m/s.

The textbooks of acoustics describe the phenomenon where wave fronts are affected by wind gradients caused by ground friction^1^, which bends the wave fronts upwards in the upwind direction, and downwards in the downwind direction. This may make the sound inaudible in the upwind direction as the sound waves bend away from the ground, creating shadow zones near the surface. However, the effect is negligible in distances shorter than about 100 m, and it thus does not explain why a human shouter perceives that it is difficult to shout against the wind.

On the other hand, acoustic measurements clearly show that in a moving medium a small stationary source emits sound with a *higher* amplitude to the upwind direction and a *lower* amplitude to the downwind direction, which is known as the acoustic convective attenuation or amplification^2^. The seeming contradiction between the physical measurements, the theory, and the common knowledge is intriguing, raising such questions as: Does the voice finally emanate with higher or lower amplitude against the upwind, and what is the mechanism behind the common perception of difficulty for the shouter to yell against the wind.

The directional pattern of the human voice in the presence of wind was addressed in earlier research of the group with measurements with real subjects, electroacoustic sources, and by simulations^3^. The results showed that the wind does not have a noticeable effect in the magnitude of radiation against upwind. Instead it was shown that the wake region of flow on the downwind side of the subject creates a wave guide effect. This result already demonstrates that the common knowledge is misleading: the sound does emanate well from shouter’s mouth against the upwind direction. Nevertheless, the work did not explain why the shouter perceived that it is somehow harder to shout against the wind.

When using their voice, humans monitor the functioning of it by listening to the sound output via the auditory system. The sound emanating from the mouth is subjected to the flow conditions in the outer world, and the effect of the flow caused by wind might have an effect to the sound arriving at the ear canals of the human. The assumption in the present study is that the wind flow around the head affects the propagation of voice to the ears of the shouter, where less sound energy is received when facing upwind, and more sound energy when facing downwind. This would explain why the shouter has an immediate perception of the difficulty of upwind shouting. The article addresses the issue with computational simulations and real measurements of the acoustical field in presence of flow field around the shouter.

The results confirm that the acoustic convective attenuation effect changes the sound pressure in ear canals explaining the perception of difficulty of upwind shouting. The magnitude of the effect depends on frequency: it is slightly higher at low frequencies than the frequency-independent analytic solution suggests, and vanishes above 3–4 kHz. The result thus suggests that it is only seemingly more difficult to shout against upwind. The sound does emanate well from a human shouter; however, the fact that the shouter hear themselves weaker creates the perception of attenuated emanation of sound against the upwind direction.

## Background

### Convective amplification and attenuation

The energy of acoustic waves emanated by a stationary source is affected by the speed and direction of the background flow. A straightforward method to quantify the effect is to have a source surrounded by microphones in static geometry, and to subject the system into conditions where the air between the devices flows. It was shown in^4^ that a plane wave radiated into a narrow tube is amplified in the upstream direction and attenuated in the downstream direction. For low-Mach flow regimes of wind speed $u$, where $u/c<0.1$ (*c* is the speed of sound 340 m/s), the pressure amplification factor *H* for the upstream versus the downstream flow was found to follow a

1 $$\begin{aligned} H=\frac{(1+u/c)^{2}}{(1-u/c)^{2}} \end{aligned}$$

relation, which agrees with theoretical results derived for a point source moving in a medium^2^[p. 723]. For example, a $u= {12}$ m/s flow would result in a 1.2 dB amplification for the wave, propagating upstream as opposed to the downstream propagating wave. The corresponding factor for 24 m/s flow is 2.5 dB. Reciprocally, a convective amplification happens when a sound source moves in a stationary medium^5^.

### Flow fields around objects in wind

When an object is placed in uniform wind, the flow circumvents the object, and the physical nature of the resulting flow depends on the size of the object and on the magnitude of the wind. Generally, on the upwind side of the object a calm region forms, while the speed of the flow on the sides of the object surpasses the speed of the uniform incoming wind. For strong enough winds, a wake region with turbulent behavior forms on the downwind side.

The behaviour of wind around a human head could be qualitatively described using a simplified case of flow around a cylinder, and it offers a good indication of the flow behaviour around more complex geometries. The flow profile around a cylinder strongly depends on the Reynolds number

2 $$\begin{aligned} \text {Re}= \frac{uD}{\nu }, \end{aligned}$$

where $u$ is the flow speed, *D* is the length scale of the system, and $\nu$ is the kinematic viscosity of the fluid. Conceptually, the numerator of the expression represents the inertial forces and the denominator—viscous forces; thus, the Reynolds number denotes the ratio between the two.

The wind speeds of interest for this work are within a range of 12–24 m/s, covering the wind strengths from strong breeze to strong gale^6^. For a circle corresponding to a cross-section of a model head, its diameter is around 0.2 m. The kinematic viscosity of air is approximately $1.5 \times 10^{-5}$ $\textrm{m}^{2}/\textrm{s}$ at 20 $^\circ$C^7^. With these values, the Reynolds number of the system under test in this study is between ${0.96 \times 10^{5}}$ and ${1.9 \times 10^{5}}$. At these wind speeds, the flow regime is categorised as subcritical or critical (critical regime starts at $\text {Re}> 10^{5}$)^8^. Therefore, flow separation and turbulent wake region are emerging features of airflow around the human head in a windy outdoor environment, which can, in turn, affect the propagation of the human voice.

In these regimes, the flow around a cylinder can be described as follows. Initially, on the incident side of the body, the flow accelerates as pressure is converted into kinetic energy. After reaching the widest cross-sectional point of the obstacle, the flow starts slowing down, and the pressure increases due to energy conservation. As the Reynolds number is relatively high, a separation boundary is formed between laminar and turbulent flow on the downwind side of the object. The flow on the laminar side of the boundary continues parallel with the general direction of the flow. At the other side of the boundary, a recirculating vortex is formed, which propagates downstream along a turbulent wake^8^. The averaged speed of the flow in turbulent wake is prominently lower than on laminar side of the boundary.

### Self-auditioning of voice production

An important part of human voice production is the ability to monitor one’s own voice in order to assess its accuracy and optimise it for the existing conditions. Such a monitoring process happens through the human hearing mechanism. The voice is transmitted along two paths from the mouth to reach the inner ear. When externally radiated from the mouth, the voice propagates through the air, reaching the outer ear. It is then registered by the hearing mechanism similarly as with any other external sound^9^, which potentially makes this propagation path subject to convective amplification or attenuation effects by flow field around the shouter. Some acoustic sound and mechanical vibration also propagates via the bones of the skull and agitates the middle and the inner ears directly. This propagation path is not affected by the flow field. The relationship between the bone- and the air-conducted parts of one’s own voice has been measured for different phonemes^10^. The vowels are the most audible phonemes, and they are thus most prominent when shouting in noisy conditions. The air-conducted part were shown to dominate the perception of vowels in most cases, while the bone-conducted sound dominated only between 1 and 2 kHz with some vowels. These findings suggest that if there are flow-field-generated effects on air-conducted path, they will most probably be audible to the shouter.

The voice of the shouter can be also reflected back from the environment, which would at least in some cases give information of the strength of emanation towards different directions. However, we argue that in an open windy environment the shouter does not usually hear the reflections, since the wind noise masks them. We thus assume that in windy conditions the perception of one’s own voice is based on immediate propagation paths from the mouth to the ears.

## Simulations and measurements

The target for the study was to quantify the effect of flow field to the sound field around a human shouter. The study was inspired by informal observations on a windy parking lot and during a bike ride. The authors noted that when uttering against the direction of the wind (i.e., upwind), one’s own voice was perceived as being ‘thinner’ (i.e., lacking low frequencies) compared to when shouting downwind. The findings motivated us to design a systematic study of the phenomenon, which included computer simulations and objective measurements.

The direct measurement of the flow field around a human in real situation is technically very challenging, and also the measurement of sound field is also subject to many sources of error. Thus, we selected to simulate the phenomenon and verify the results with a limited number of acoustical measurements from a real source in flow fields.

When facing either upwind or downwind, the ears of the shouter are in the region of the surface of the head where higher flow speeds are observed. Simulating the acoustic field in flow around geometrically complex objects, such as a human head, is prone to simulation errors due to many reasons. We are assuming that the effect can be researched with geometrically simpler objects, since the flow causes similar speed-up regions around any object with a similar form and size. An infinitely long cylinder with radius of 10 cm was selected to be the modeled geometry. The cylinder was selected, since the head with the torso could be approximated by a section of a cylinder. The usage of a cylinder is also supported by a study presented in^11^, where the flow effects around a walking human body were simulated using a section of a cylinder. Its authors report a relatively good match in the produced flow field with the simplified geometry to the one produced by a detailed model of human body.

The estimate of convective amplification as a function of frequency ${\tilde{H}}(f)$ can then be computed from the simulated sound field as

3 $$\begin{aligned} {\tilde{H}}(f) = \frac{{\tilde{X}}_{\textrm{uw}}(f)}{{\tilde{X}}_{\textrm{dw}}(f)}, \end{aligned}$$

where ${\tilde{X}}_{\textrm{uw}}$ and ${\tilde{X}}_{\textrm{dw}}$ are the simulated ear canal magnitude spectra in upwind and downwind conditions, respectively.

A physical measurement was also conducted to verify the cylinder modeling results. A simplified arrangement was used with an artificial speaker and microphones placed around a cylinder with radius of 10cm, and the sound recorded in a set of different wind speeds and directions. The wind was generated by installing the device on top of a car, and by driving on an airport runway. The estimation of conductive amplification ${\hat{H}}(f)$ is then computed as

4 $$\begin{aligned} {\hat{H}}(f) = \frac{{\hat{X}}_{\textrm{uw}}(f)}{{\hat{X}}_{\textrm{dw}}(f)}, \end{aligned}$$

where ${\hat{X}}_{\textrm{uw}}$ and ${\hat{X}}_{\textrm{dw}}$ are the magnitude spectra of sound measured at the positions of ears in upwind and downwind uttering conditions, respectively.

## Results

**Figure 1:**
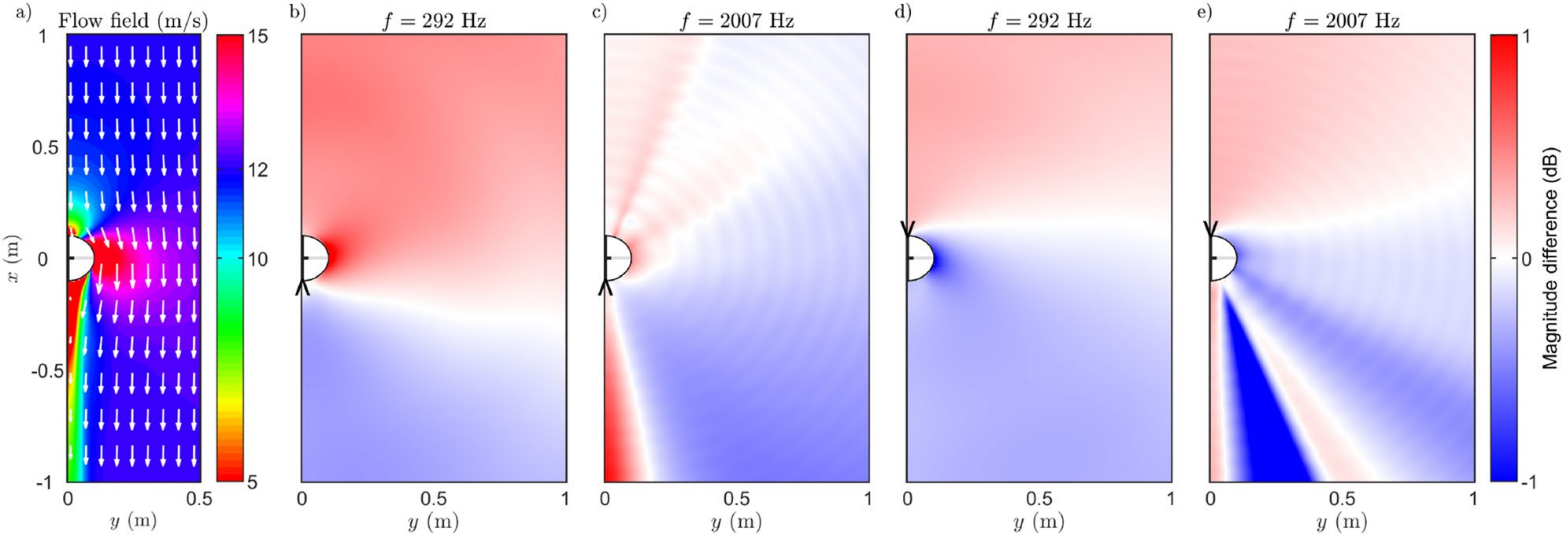
(**a**) The magnitude of simulated flow field $\left( u_x^2+u_y^2\right) ^{1/2}$ with 12 m/s inlet wind. (**b**)–(**c**) Relative sound field when the source is facing downwind compared to the static condition at different frequencies. (**d**)–(**e**) Relative sound field when the source is facing upwind compared to the static condition at different frequencies. Symbol $\vee$ or $\wedge$ denotes the source location.

The simulated flow field and the pressure field around a cylinder are presented in Fig. 1 for four different simulations with different source frequencies and with different directions of the mouth relative the flow. The simulated flow field in Fig. 1a visualize the effect discussed earlier in this article: steady wind causes increased flow speed on the sides of the cylinder, and on the downwind side of the head a wake region with lower velocity of flow forms.

**Figure 2:**
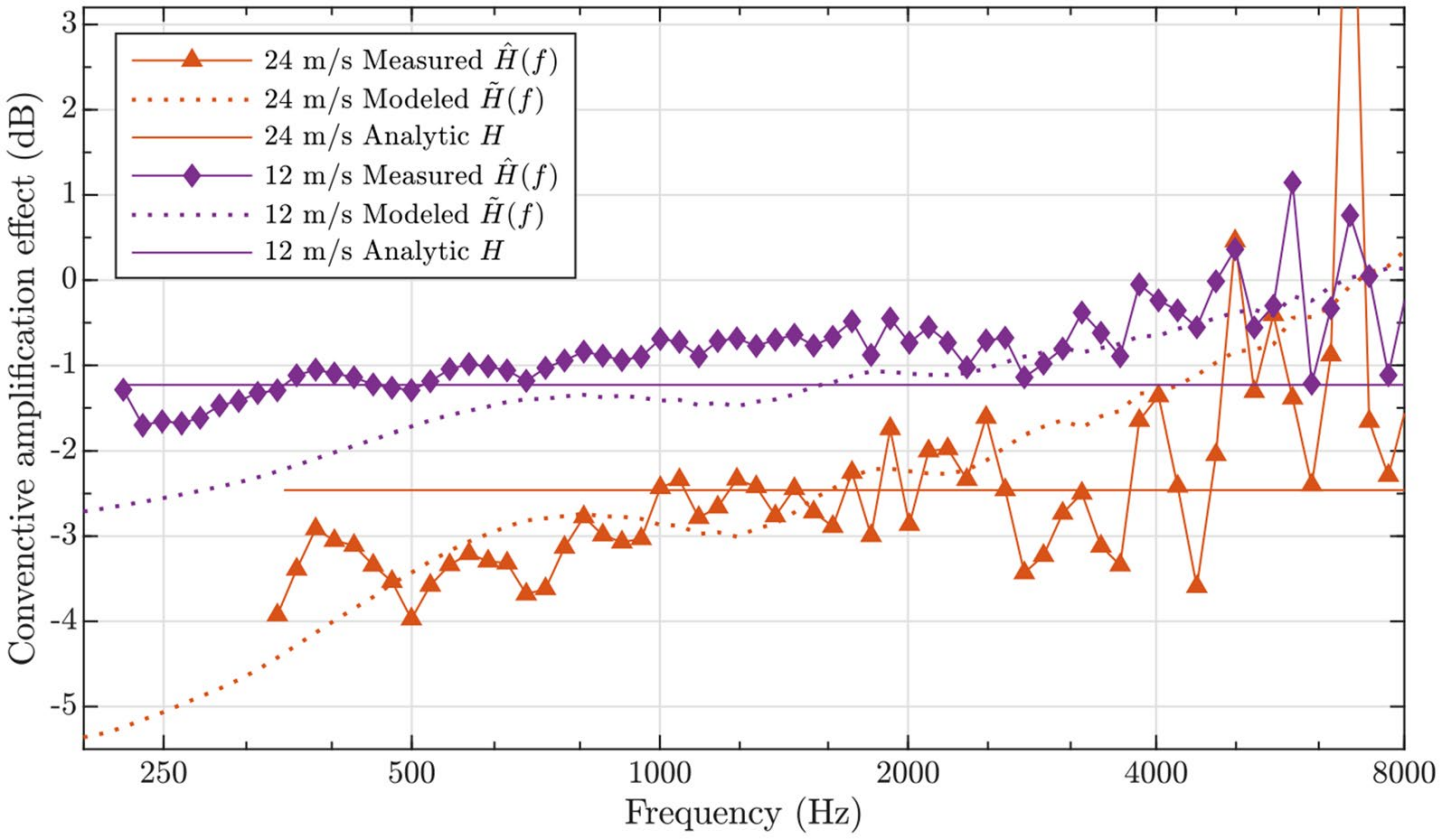
The change of sound pressure level in the vicinity of ear canals in upwind shouting vs. downwind shouting. The convective effect is estimated using measurements, multiphysics simulations, and with an analytic solution (Eq. 1) valid for point sources in free field. The effect is shown in decibel scale computed by taking $20 \log {(\cdot )}$ on estimated or measured value. Values lower than zero imply lower sound pressure level at ears in the case of upwind shouting than in the case of downwind shouting. A cylindrical object was placed in static air flow, with sound source either facing upwind or downwind.

The effect of convective amplification can be seen in simulated sound fields presented in Fig. 1b–e. In all cases the sound pressure is higher on the upwind side of the source and lower on the downwind side, disregarding the wake region, for both upwind and downwind uttering directions. The convective amplification effect manifests as a boundary between higher and lower sound pressure regions, which meet the position of the mouth and extend horizontally in the figures. The orientation of the boundary depends on frequency and orientation of the source in the flow. The results also show the known effect that the wake region acts as a wave guide for higher frequencies^3^.

The main assumption in the article concerns the self-auditioning of the voice, and thus the change of sound pressure in the positions of ear canals caused by wind conditions is of the main interest. In Fig. 1d,e, the mouth is directed against the upwind, and there is an area of reduced sound pressure near the ear positions, visible as darker blue color. Vice versa, in Figs. 1b,c, the mouth is directed against the downwind increasing the pressure next to the ears as shown by darker red colors. This result thus fits the assumption that the flow field around the ear makes one’s own voice less audible when yelling against upwind. It is assumed that the minor ridges following equiphase circles around the source visible in Fig. 1c,e are caused by numerical errors in simulations.

The convective amplification effect estimated for acoustical path from the mouth to the ear in the upwind and downwind conditions using measurements, simulations and analytic expression are presented in Fig. 2 for two wind speeds. The analytic result is computed using Eq. (1) with the knowledge that the ears are on the downwind side of the mouth, producing convective attenuation effect. Both the simulated and measured curves exhibit similar dependency with frequency, at lower frequencies the effect is larger and decreases slowly with frequency. All the results thus suggest that the sound pressure level in the ear canals of the shouter will be lower in the upwind shouting case than in the downwind shouting case. Above 4 kHz the measured result is unstable with frequency. The measured and simulated effects match relatively well with each other below 2 kHz for 24 m/s case. A similar dependency on frequency can be observed at 12 m/s, although the measured results seem to be biased towards zero by 0.5 dB to 1 dB from modeled results. The discrepancy may have been caused by less stable measurement conditions at lower wind speeds, since background wind fluctuations have a stronger relative impact on the direction and magnitude of airflow during the measurement. The modeled and simulated frequency-dependent results are in 0.5 dB range from the analytic frequency-independent solution approximately in 500 Hz to 1000 Hz in 12 m/s case, and in 1000 Hz to 2000 Hz in 24 m/s case.

## Discussion

The results from both measurements and simulations as well as the analytic equation all suggest that the perceived difficulty of shouting upwind would be at least partially explained by the convective attenuation effect. Perhaps a limitation of the study is the usage of simplified geometry in both measurements and simulations. Using a binaural mannequin with a mouth simulator would have provided better correspondence to human geometry, however, both modeling and measurements with it had several technical difficulties. The measurements require relatively high sound pressure levels to overcome the noise caused by the flow, which is not achievable with devices available. In modeling, the use of human-like geometry in both flow field and acoustic field was not solvable with current computational resources. For these reasons, the simulations and the measurements were conducted with simplified geometries of a human subject and the validity of the approach is discussed below.

At frequencies below 4 kHz, the wavelength of sound is larger than 8 cm, and at 100 Hz, the lowest frequencies analysed, the wavelengths are of the order of meters. Thus, fine details of geometric models are very unlikely to have an effect on acoustic modeling results. However, it is acknowledged that relatively small geometric details, such as pinnae, may cause prominent changes in the formation of eddies in the flow around the head. The main observed effect of sound attenuation along the upwind path from the mouth to the ears may be reduced by any finer head features that would be able to decrease or reverse the flow along the direct path between the mouth and the ear canal. However, the most prominent parts of the pinnae, excluded from the current models, are positioned behind the transmission path. They would contribute to an increase in turbulence in the wake region behind the head, but the effect on flow in the upstream direction would be minor. Therefore, a more detailed head geometry is unlikely to have a major effect on the modelled and measured results of the convective amplification phenomenon. We are thus assuming that the simulations and the measurements presented in the article represent the flow field around a human shouting in windy conditions with high enough accuracy for the conclusions presented below.

##  Conclusions

The effect and existence of convective attenuation during the self-auditioning of the human shouter in the upwind and the downwind shouting directions was researched by computational modeling and real measurements of cylindrical models of the shouter. The theory of convective attenuation phenomena suggests that, since the ear canals of the shouter are on the downwind side of the mouth when shouting upwind, the amplitude of sound waves arriving at the ear canals should be reduced, and vice versa for downwind shouting. The modeling studies and the measurements consistently show the presence of the convective amplification/attenuation effect under 4 kHz. We also show that the presence of the subject in the flow increase the effect for upwind and downwind shouting at low frequencies, which is caused by the speed-up of the flow on the sides of the head of the subject. The convective attenuation effect thus explains the perceived difficulty and the change in the perceived timbre (sound colour) of one’s own voice when shouting upwind.

## Methods

### Simulations

**Figure 3:**
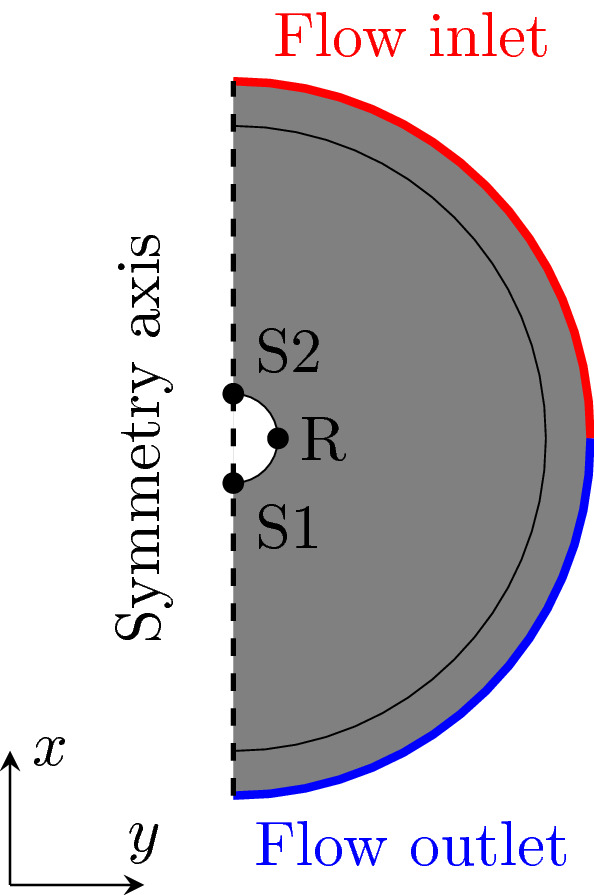
Schematic geometry of the simulation. In the model, upwind and downwind scenario is modelled by moving the the source position between S1 (upwind) and S2 (downwind). The outermost layer of the circular domain is defined as PML for the acoustic simulation.

The modelling was undertaken in COMSOL Multiphysics 6.0. The schematic problem geometry is shown in Fig. 3. In the model, the geometry is assumed to be 2D, with a circle of 10 cm radius in the center of the region. The outer domain boundary is at 2 m radius from the centre. The outer layer of width 50 cm was dedicated to the perfectly matched layer (PML) in the acoustics simulation.

The simulation was divided into three steps. Firstly, the turbulent flow was computed for a chosen wind speed and direction using a CFD model mesh. Then the background flow fields, namely pressure, velocity, and turbulent viscosity, were mapped from the CFD solution to the acoustics mesh. Finally, the acoustics simulation was run using the acoustics mesh with background flow fields. A parametric sweep interface ran the model with different combinations of wind speeds and directions. The sections below present more detailed information on the model setup.

#### CFD model setup

The Shear Stress Transport (SST) turbulent flow physics interface was used to model the fluid flow. The flow was modelled as incompressible. For the applied model, the equations for the turbulent kinetic energy *k* and specific dissipation rate $\omega$ can be written as

5 $$\begin{aligned} \rho (\textbf{u}\cdot \nabla )k= & {} \nabla \cdot \left[ \left( \mu +\mu _{\text {T}}\sigma _{\text {k}}\right) \nabla k\right] + P - \beta _0^*\rho \omega k, \end{aligned}$$

6 $$\begin{aligned} \rho (\textbf{u}\cdot \nabla )\omega= & {} \nabla \cdot \left[ \left( \mu +\mu _{\text {T}}\sigma _{\omega }\right) \nabla \omega \right] + \frac{\gamma ^*\rho }{\mu _{\text {T}}}P - \beta \rho \omega ^2 + 2\left( 1-f_{v1}\right) \frac{\sigma _{\omega 2}\rho }{\omega }\nabla k\cdot \nabla \omega , \end{aligned}$$

where

7 $$\begin{aligned} P = \min \left( P_k, 10\rho \beta _0^* \omega k\right) \quad \text{ and }\quad P_k = \mu _{\text {T}}\left[ \nabla \textbf{u}: \left( \nabla \textbf{u}+ \left( \nabla \textbf{u}\right) ^\top \right) \right] . \end{aligned}$$

In (5)–(7), $\textbf{u}$ is the velocity field, $\mu$ is the dynamic viscosity, and $\rho$ is the density. In addition, $f_{v1}$ is the interpolation function, $\mu _{\text {T}}$ is the turbulent viscosity, and $\sigma _k, \sigma _\omega , \beta , \beta _0^*, \gamma ^*$ are the model constants. The properties of air at 1 atm reference pressure and 20 $^\circ$C temperature were used in all simulations.

No-slip wall boundary condition, that sets the velocity field $\textbf{u}=0$, was assigned to the surface of the target object. The outer circular boundaries were split in half: one quarter-circle served as an inlet, the other as an outlet (see Fig. 3). The inlet was assigned with a velocity boundary condition, $\textbf{u}=\textbf{u}_0$, where the velocity of incoming air was defined to be parallel to the *x*-axis, i.e. the *y*-component of $\textbf{u}_0$ was set to zero. Inlet velocities 12 m/s and 24 m/s as well as 0 m/s (stationary case) were used. The outlet was modeled as a pressure-outflow with suppressed backflow option activated. In equation form, this boundary condition reads

8 $$\begin{aligned} \left( -p\textbf{I}+ \mu \left( \nabla \textbf{u}+ \left( \nabla \textbf{u}\right) ^\top \right) \right) n= & {} {\hat{p}}_0n, \end{aligned}$$

9 $$\begin{aligned} {\hat{p}}_0n\le & {} 0, \end{aligned}$$

where *p* denotes pressure, $\textbf{I}$ is the identity matrix and *n* the boundary normal direction. The model is symmetric (also with respect to flow direction), so the inlet and outlet positions were fixed, and different wind directions were modelled by changing the position of the source in the acoustics simulation.

A stationary solver with wall-distance initialisation (required for the turbulence model) was used to simulate the velocity, pressure, and turbulent viscosity fields. These variables were then mapped to the acoustics mesh and used as background parameters in the acoustics study.

Linear basis functions were selected for the CFD simulations. The element size for the computational grid was selected automatically by COMSOL. On the no-slip wall, a boundary layer mesh with 6 layers and 1.2 stretching factor was applied. For the mesh resolution criteria, we selected the ‘extra fine’ option. More detailed discussion on the CFD model can be found in the theoretical publication^12^ and the COMSOL manual^13^.

#### Acoustics model setup

A linearised Navier-Stokes interface was used to simulate the acoustic wave propagation. For the wave simulations, background pressure $p_b$, mean flow $\textbf{u}_b,$ and turbulent viscosity $\mu _{\text {T}}$ were mapped from the CFD solution using the COMSOL build-in feature ‘Background Fluid Flow Coupling’. The governing equations for acoustics simulation are as follows

10 $$\begin{aligned} i\omega _{\text {a}}\rho _t + \nabla \cdot \left( \rho _t \textbf{u}_b+\rho _b\textbf{u}_t\right)= & {} M, \end{aligned}$$

11 $$\begin{aligned} \rho _b\left( i\omega _{\text {a}}\textbf{u}_t + \left( \textbf{u}_t\cdot \nabla \right) \textbf{u}_b + \left( \textbf{u}_b\cdot \nabla \right) \textbf{u}_t \right) + \rho _t\left( \textbf{u}_b\cdot \nabla \right) \textbf{u}_b= & {} \nabla \cdot \sigma -\textbf{u}_b M, \end{aligned}$$

12 $$\begin{aligned} \rho _b C_p\left( i\omega _{\text {a}}T_t+\textbf{u}_b\cdot \nabla T_t\right) -\alpha _pT_b\left( i\omega _{\text {a}}p_t+\textbf{u}_t\cdot \nabla p_b+\textbf{u}_b\cdot \nabla p_t\right) -\alpha _p T_t\left( \textbf{u}_b\cdot \nabla p_b\right)= & {} \nabla \cdot \left( k_a\nabla T_t\right) , \end{aligned}$$

where

13 $$\begin{aligned} \sigma= & {} -p_t\textbf{I}+ \mu _t\left( \nabla u_t+\left( \nabla u_t\right) ^\top \right) +\left( \mu _B-\frac{2}{3}\mu _t\right) \left( \nabla \cdot u_t\right) \textbf{I}, \end{aligned}$$

14 $$\begin{aligned} \rho _t= & {} \rho _b\left( \beta _Tp_r - \alpha _pT_t\right) . \end{aligned}$$

In (10)–(14), $\omega _{\text {a}}$ is the angular frequency and $p_t, \textbf{u}_t,$ and $T_t$ are the total acoustic perturbations to the pressure, velocity, and temperature, respectively. In addition, coefficient of thermal expansion $\alpha _p$ and isothermal compressibility $\beta _T$ are computed from

$$\begin{aligned} \alpha _p =\frac{1}{c_b}\sqrt{\frac{C_p(\gamma -1)}{T_b}} \quad \text{ and }\quad \beta _T =\frac{1}{\rho _b}\frac{\gamma }{c_b^2}, \end{aligned}$$

where $c_b$ is the speed of sound, $C_p$ is the heat capacity, $\gamma$ is the ratio of specific heats, $T_b$ is the air temperature, and $\rho _b$ is the density. Note that parameters $c_b, C_p$ depend on the temperature $T_b$ while $\rho _b$ depends on $T_b$ and background pressure $\rho _b$. Finally, $\mu _B$ introduced in Eq. (13) denotes the bulk viscosity, $\mu _t=\mu +\mu _{\text {T}}$ is the total effective viscosity, and $k_a$ introduced in Eq. (11) denotes the thermal conductivity.

The circle was modelled as a slip wall $n\cdot \textbf{u}_t=0$ with an adiabatic thermal condition $-n\cdot (-k_a\nabla T_t)=0$. The sound source was modelled as a domain mass source. A Gaussian distribution described the location and the size of the source *M*. Its peak was defined at a boundary point (letters S1 and S2 in Fig. 3). The spatial source spread (standard deviation) in both models was 1cm. For a more detailed and complete discussion about the applied acoustic model and its implementation, we refer to COMSOL Acoustics Module User’s Guide^14^.

A domain probe recorded the simulated pressure data at the receiver position (letter R in Fig. 3). The simulations modelled logarithmically spaced frequencies from 150 up to 8000 Hz. The specific frequencies modelled correspond to the frequencies used in the measurements, described below.

Linear basis functions were used to discretise all the field variables (pressure, velocity, and temperature). As for the grid density, requirement was set to ten elements per wavelength at minimum. The same computational grid was used for all frequencies. The solver configurations used in the studies were set automatically by COMSOL. COMSOL Acoustics Module User’s Guide^14^ and COMSOL support were consulted to achieve the best stability and performance of the models. The results obtained from the simulations were analysed and visualized in MATLAB.

### Measurements

The physical experiment aimed to create a controlled environment and a reproducible procedure, with unaccounted factors minimised. A measurement methodology, proposed in^3^, was adapted for this experiment: a vehicle with the measurement rig on the roof was driven at a constant speed in a free field. Due to the relativity of motion, the movement of the source replicated the wind blowing in the opposite direction. Although it was difficult to control the exact wind speed and direction in the open air, anemometers monitored and recorded the wind speed while a visual wind vane informed about the wind direction during the experiment. The measurement segments were then sifted in the post-processing stage based on the stability of the wind to minimise the effect of wind variation on the final results.

The 2D domain used for the simulations influenced the shape of the measurement rig. A long cylinder of a 20 cm diameter was used to imitate the flow around a cross-section of the human head. Consequently, the discussion of wind effects is limited to horizontal flows and symmetric circular head geometry. The turbulent flow and sound propagation from point sources behave differently in the 2D model compared to the physical 3D world (point source in 2D corresponds to a line source of infinite length in 3D). However, the study was mainly interested in relative differences between the responses for different wind conditions, while the discrepancies in turbulent effects were considered minor compared to the main effect on sound pressure from boundary layer flow gradients. Although a 2D simulation of a circle corresponds to a cylinder of infinite length, the measurement rig was limited to 1.5 m height due to technical constraints. The speaker was installed mid-height (0.75 m) to minimise any potential effects from the cylinder top and the roof of the van.

Table 1 reports the equipment used for the measurements. A high sensitivity compression driver was used as a speaker. Omnidirectional pressure microphones were fit on the outside of the cylinder at the same height as the speaker (0.75 m) 90 $^\circ$ away from the ‘mouth’ to represent ear positions. A control microphone was also placed below the driver to monitor the sound at the position of the ‘mouth’. The inside of the pipe was filled with absorptive foam material to eliminate resonances of the enclosure. Microphone windshields were used to reduce the wind-induced noise on the microphone. Generally, the airflow might be slowed down, and the turbulence effects increased within and around the windshields due to the change in local surface roughness. However, this influence on the measurements was deemed minor: the main wind effect on sound was assumed to develop along the sound propagation path from the speaker to the microphone, whereas the windshields affected the airflow primarily at the microphone position and behind it. Furthermore, only relative sound level differences were studied, so any potential biases, which affected all the measurements equally, were eliminated in the analysis stage.

A multitone signal was chosen for the measurements due to its characteristic signature in the spectrum, robustness with time-variant systems and broad frequency range. It was composed of a hundred logarithmically-spaced sine tones of randomised phases within the frequency range of 50–10000 Hz. An FFT window of $2^{16}$ samples (which equates to approx. 1.365 s signal length for a 48000 Hz sample rate used) was chosen, and the tone frequencies were matched to the corresponding FFT frequency bins. Five-second sound segments were used with 2.5 s silent gaps in between so that the background noise could be captured independently of the signal and signal-to-noise ratio (SNR) estimated. To achieve the highest possible dynamic range and SNR, a constraint on the crest factor (CF, defined as the ratio between the signal peak and RMS values) of the signal was imposed while randomising the phases of the sine components.

**Figure 4:**
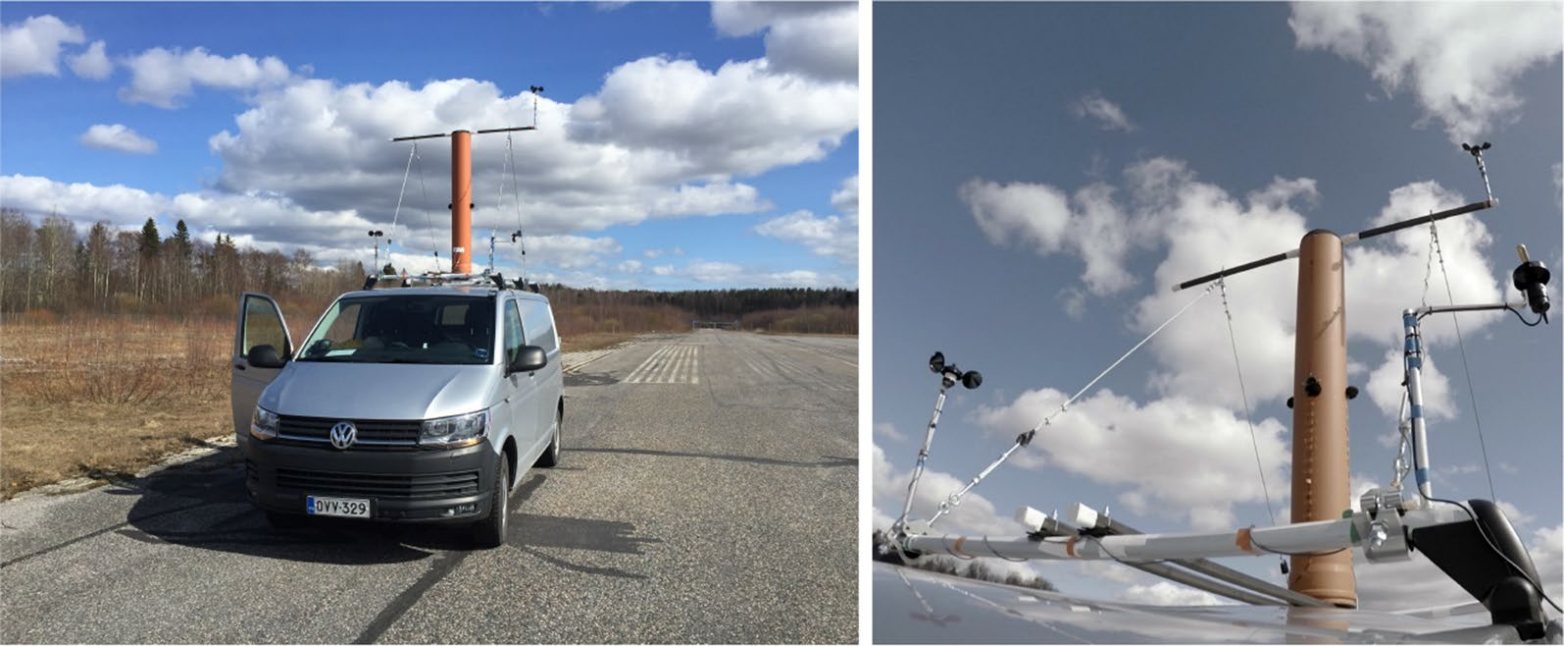
Still images of the measurement rig on the roof of the van and GoPro footage of 0 $^\circ$ (upwind) measurement setup.

The measurement rig was secured on the roof by wire ropes on either side of the roof bars, as seen in Fig. 4. Different wind directions to the speaker were imitated by rotating the cylinder. The measured directions were 0 $^\circ$ (the loudspeaker facing the driving direction to imitate upwind condition, shown in the right picture) and 180 $^\circ$ (downwind condition, visible in the left picture). Two anemometers were installed on the roof: one used to monitor the wind speed live while driving, another one recorded via the audio interface. The wind vane was also installed, and its position recorded using a video camera, attached to the front windscreen.

**Table 1:** Summary of the equipment used in the measurements.

Item	Model	Notes
Driver	BMS 4594ND-MID	1.4′′ two-way compression driver; only the mid-range driver was used
Microphones	DPA 4061	Omnidirectional microphones; max peak SPL =144 dB
Windshields	Rycote Windjammers	Used on top of foam windscreens.
Amplifier	S.M.S.L. SA-50	50 W class D amplifier.
Audio interface	RME UFX+	
Recording software	Reaper DAW	
Cup anemometers	Inspeed Vortex	Cateye Velo8 cyclocomputer used to monitor wind speed. Data recorded via audio interface.
Wind vane	Inspeed e-Vane	Direction inspected visually via GoPro cameras

#### Measurement conditions and procedure

The experiment was conducted in Helsinki-Malmi airport. A 500m section of the runaway, which ran east-to-west, was used. The direction of the drive was kept constant throughout the measurements.

The sky on the day of the measurements was partly cloudy with some sunny spells. The temperature was 5 $^\circ$C in the morning at the start of the measurements and increased to 12 $^\circ$C in the afternoon. Relative humidity decreased from 70% in the morning to 36% in the afternoon. The ambient pressure rose from 102.0 to 102.2 kPa.

Amplifier level and microphone gains in the audio interface were fixed throughout the measurement. The amplifier gain dial was set slightly over half the maximum to avoid distortions from the amplifier and the driver. The stability of the sound source throughout the measurements was verified by measuring SPL at the position of the side microphone with a calibrated Class 1 Sinus Tango sound level meter. 15-second-long measurements of the multitone signal were recorded after each rotation of the speaker while the car was stationary. For all three directional setups, the recorded A-weighted equivalent SPL was $L_{\textrm{Aeq}} = {114}\,{\textrm{dB}}$. Measured background sound level was $L_{\textrm{Aeq}} ={49}\,{\textrm{dB}}$.

For each source direction, measurements were conducted in 12 m/s and 24 m/s wind speeds. Based on the live anemometer readings, the driving speed was adjusted to correct a mismatch between the wind speed and the driving speed due to ambient wind. Each drive for a specific wind direction and speed combination was repeated three times. Before and after every drive (i.e. on both sides of the runaway), a single 5 s multitone burst, surrounded by silence, was recorded. These stationary measurements were later used to calculate the difference between the frequency response, measured in the wind versus no-wind conditions. Every measurement conducted while driving was composed of a set of five-second multitone signals separated by silent gaps. The number of them differed depending on the drive speed, which limited the duration of each drive.

#### Data analysis

The most stable measurement segments were selected based on the wind measurement recordings from the anemometers. A moving window of $2^{16}$ samples (corresponding to the multitone FFT size) selected non-overlapping measurement periods with the standard deviation of instantaneous speeds below 0.4 m/s. The stationary measurements were restricted by 2.5 m/s instantaneous wind speed limit instead. A set of background noise segments of the same length, recorded just before or after each signal measurement, were also selected to represent the existing noise floor.

An FFT was then computed for each measurement segment. There were multiple segments chosen from each driving condition. Therefore, the mean magnitude response for each condition was calculated using incoherent averaging of the magnitude spectra to reduce noise variance^15^. The same averaging process used for signal segments was also implemented for the background noise samples. The noise floor for each averaged measurement was then estimated by finding a peak envelope. A 20 dB SNR criterion was imposed on all the multitone peaks: if the magnitude of the peak was lower than 20 dB above the noise floor at that frequency, the peak was discarded from further analysis.

The stationary measurements taken on both sides of the runaway were analysed and averaged using the same techniques as the measurements taken while driving. The mean magnitude response for each wind velocity was compared to its corresponding stationary response: the decibel levels of the measurements in the wind were subtracted from those in no-wind conditions. Only the magnitudes of multitone frequencies, which mutually satisfied the SNR criteria, were used. The magnitude responses in the wind were compared to their corresponding no-wind responses measured around the same time to minimise the biases of each measurement caused by the ambient weather and noise conditions. The obtained relative magnitude responses for each wind condition were ultimately compared against each other to establish the effect of wind on the recorded magnitude responses.

#### Data reliability

Table 2 presents means and standard deviations of wind speeds, which were calculated from the measurement segments, selected for the mean magnitude response calculations. It reports the wind data for each combination of the speaker direction and the driving speed measured. The number of measurement segments used in the analysis differed for each scenario (indicated in column $N$ of the table). This variation was caused by the wind stability criteria imposed in the data analysis stage and a shorter measurement length when higher driving speeds were used. Each driving condition had a set of stationary measurements associated with it, taken immediately before and after each drive, so averaged values of each stationary wind measurements are reported alongside the driving scenarios in the same table. Unlike in the driving case, where the primary wind direction was assumed to be reasonably steady against the drive direction, wind direction during the stationary measurements was unpredictable. The speed averaging process did not take the directionality into account. Therefore, direct wind speed comparisons between the driving and the associated stationary conditions cannot be made. Instead, stationary wind measurements indicate the prevailing wind condition and possible biases of the measurements.

**Table 2:** Summary of mean measured wind speeds and standard deviations over the measurement segments used in the magnitude response analysis. Each averaging was done over $N$  segments without taking the wind direction into account.

Speaker direction	Driving			Stationary		
	$\overline{u}$ (m/s)	$\sigma _{u}$ (m/s)	$N$	$\overline{u}$ (m/s)	$\sigma _{u}$ (m/s)	$N$
0 $^\circ$ (upwind)	11.7	0.7	27	2.4	0.0	2
	23.3	0.8	8	0.8	0.8	9
180 $^\circ$ (downwind)	12.0	1.0	22	1.9	0.4	5
	23.5	0.4	4	1.4	1.0	4

Mean wind speeds of the chosen driving segments agree with the desired and modelled wind speeds of 12 m/s and 24 m/s within 0.5 m/s range. Their standard deviations are within the range of 0.4–1.0 m/s. The wind speeds of the stationary measurements reach a maximum of 2.4 m/s, which is just below the limit of 2.5 m/s, imposed on the stationary measurements in the data analysis stage. The standard deviation of the stationary measurements is less than or equal to 1.0 m/s. On average, the prevailing background wind in the downwind measurements is higher than in the upwind ones. A visual review of the recorded footage of the weather vane revealed that the dominant wind in the first half of the measurements (0$^\circ$ direction) was westerly and north-westerly. In the second half of the measurements (180$^\circ$ direction), the wind changed to a predominantly south-westerly and south wind (the direction of the drive was east-to-west).

**Figure 5:**
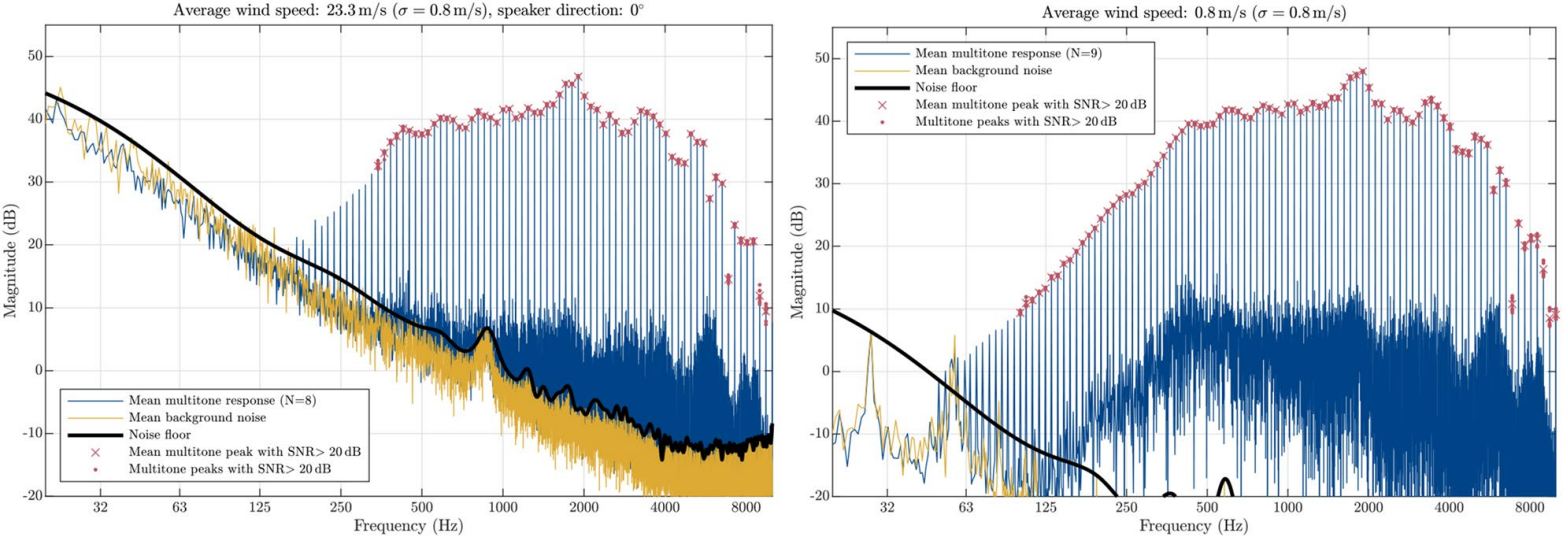
An example of mean measured magnitude responses and background noise floor for upwind direction and its associated no-wind response. Red dots indicate the spread of individual peaks, used in the averaging process and the crosses mark their mean value.

Figure 5 shows an example of mean magnitude response for the measurement in the wind and an associated mean stationary measurement. Mean background noise responses and the estimated noise floors are also plotted on the figures. The multitone peaks, which satisfy the 20 dB SNR criteria, are marked by red crosses. Additionally, red dots represent the data used in the averaging process and indicate the spread of the measurements.

Examples of measured magnitude responses in wind and stationary conditions plotted in Fig. 5 represent one of the two measurement scenarios with the highest wind speed and therefore the highest recorded background noise profile. The envelope of the background noise across all the measurements was of a similar low-pass nature with an approximate negative slope of 10 dB per octave but lower in overall level for slower driving speeds. Due to the high output power of the compression driver and the low-pass nature of the wind-induced noise, multitone peaks as low as 350 Hz achieved the desired SNR criteria even in the highest wind speeds measured. At the highest wind speeds, the background noise had a peak in the magnitude response around 800 Hz, which was heard as a whistling sound during the measurements. However, the magnitude response of the speaker in this frequency range highly surpassed the desired SNR, so the whistle did not affect the measurement results.

## Data Availability

The datasets used and analysed during the current study available from the corresponding author on reasonable request.
